# Editorial: Microplastics and Microorganisms in the Environment

**DOI:** 10.3389/fmicb.2022.947286

**Published:** 2022-06-28

**Authors:** Xianhua Liu, J. Paul Chen, Lei Wang, Zongze Shao, Xiang Xiao

**Affiliations:** ^1^School of Environmental Science and Engineering, Tianjin University, Tianjin, China; ^2^Department of Civil and Environmental Engineering, National University of Singapore, Singapore, Singapore; ^3^College of Environmental Science and Engineering, Nankai University, Tianjin, China; ^4^Third Research Institute of the Ministry of Natural Resources, Xiamen, China; ^5^School of Oceanography, Shanghai Jiao Tong University, Shanghai, China

**Keywords:** microplastics, microorganisms, environment, pollution, interaction

Microplastic pollution is a global challenge. Microplastics can persist in different areas of the environment; however, their long-term impact on environmental health is still largely unknown (Wang et al., 2020, 2021). Recent studies have shown that microorganisms may play essential roles in the dispersal and transformation of microplastics (Wang et al., 2019; Zhang et al., 2021; Peng et al., 2022). Hence, it is crucial to study the interactions between microorganisms and microplastics so that an understanding of their environmental impact can better be obtained and effective control measures can be designed and adopted.

The eight papers in this virtual special issue (VSI) cover a wide range of microbiological research aspects on microplastics in different environments from mollisols of northeast China to a small headwater stream in Germany as illustrated in Figure 1A. This collection provides a snapshot of current research interests and findings in the field. The papers can be divided into four topics according to their contents: microplastics in the soil environment, the microbial community of biofilm, the impact of microplastics on organisms, and interactions of microplastics with such contaminants as heavy metals (Figure 1B). In addition to the keywords used in the VSI such as microplastics, the terms frequently used are soil, biofilm, treatment, exposure, effect, surface, compare, heavy metal, increase, high, abundance, and microorganism (Figure 1C). They show the unique characteristics of this VSI, and their distribution among four topics is shown in Figure 1D.

**Figure 1:**
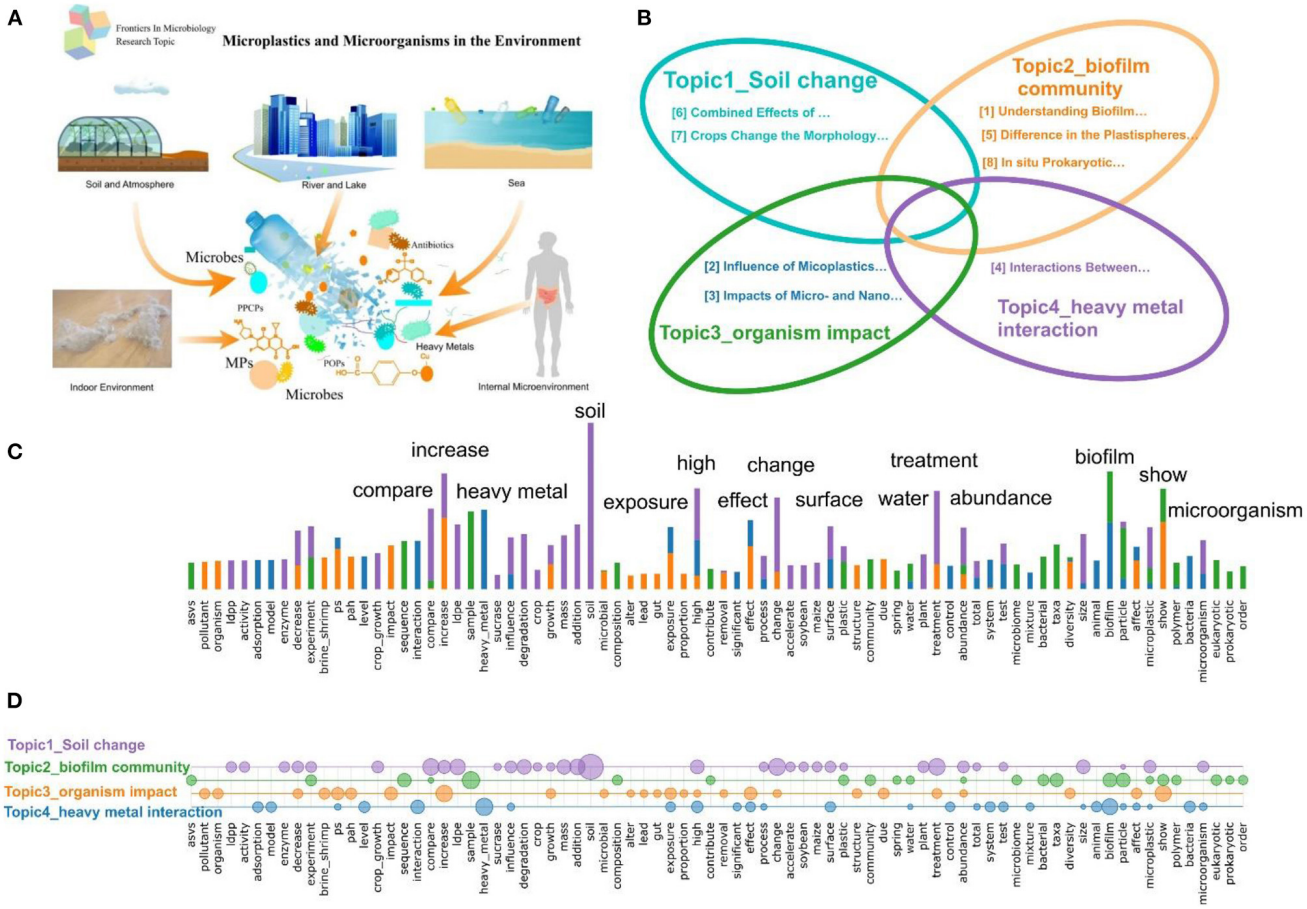
**(A)** Schematic of the relationship between microplastics and microorganisms in the environment; **(B)** The four topics of this VSI; **(C)** Terms with high frequency in this VSI; **(D)** The distribution of some high-frequency terms among the four topics. The circle area represents the term frequency, and different colors represent different topics.

The first topic addresses the microplastics in the soil environment. Ren et al. demonstrated the existence of microplastics in soil can alter the effect of popular remediation technologies. There are two possible reasons: the first one is the adsorption and enrichment effect of microplastics on organic pollutants, and the second is the screening effect of microplastics on specific pollutant-degrading microorganisms. Through the studies of several key parameters, such as soil amendment, microplastic types, and the effect of microplastic content, Wang et al. reported that crop growth can accelerate the microplastic's change in the soil. The growth of crops changed the morphology, functional groups, total mass, and abundance ratio of microplastics of different sizes, which may be attributed to the enzymes in the soil, especially around the rhizosphere.

The second topic of this VSI provides some new perspectives on plastic biofilm. Firstly, the inhabitation of a plastic surface by biofilm can undergo various physiochemical transformations, resulting in changing particle capacity to sorb chemicals and aggregate with other particulates. The ecological and microbe-microbe interactions in plastic biofilms may favor some taxa with concomitant effects on the consumers (Gorokhova et al.). Secondly, the microbial communities in the plastisphere of biodegradable plastics and non-biodegradable plastics behaved differently (Peng et al.). It was observed that the content of the plastisphere biomass on the surface of biodegradable plastics was higher than that of non-biodegradable plastics. Therefore, the potential environmental risks of degradable microplastics should not be downplayed due to their higher potential in the enrichment of antibiotic resistance genes and pathogens. Thirdly, a strong correlation of polymer-specific clustering in prokaryotic and eukaryotic communities was discovered in the natural environment (Weig et al.). Different microplastic particles were colonized by different biofilm communities, however, similar percentages of pathogenic bacteria were identified over a broad range of polymer and quartz surfaces, suggesting that these potentially harmful microorganisms can colonize many surfaces.

The third topic regards the toxicological effects of microplastics. In a long-term exposure experiment, it was found that microplastic exposure significantly reduced the growth rate of brine shrimp, and different polymer types had different effects (Li et al.). Ingestion of microplastics remarkably changes intestinal microbiota. The relationship between growth inhibition and changes in intestinal microbiota deserves further study. Microplastics may also have an impact on photosynthetic organisms (Li et al.). Photosynthetic organisms are an extremely important part of the ecosystem and the main producer of organic carbon. The effects of micro- and nanoplastics (MNPs) on the photosynthesis of aquatic and terrestrial photosynthetic organisms have been widely reported. These facts show that MNPs can pose potential risks to ecosystem health and food safety. However, the understanding of their impact on the environment and their action mechanisms are still very limited.

The fourth topic is on the interactions of microplastics and the coexisting pollutants in the natural environment. As a vital biological component in the ecosystem, microorganisms play an important role in regulating the interactions between microplastics and other environmental pollutants (Liu et al.). For example, under the stress of heavy metals, microbial cells in biofilms can produce more extracellular polymeric substances (EPS) to protect themselves from a harsh environment. The EPS can absorb heavy metals and inhibit the diffusion of heavy metals into the matrix, by which the concentrations of heavy metals decrease to sublethal concentrations. The survival of exposed microbes thus develops the ability of tolerance or resistance to heavy metals. How these selection processes influence the fate of MPs and heavy metals are still unknown. Research on the combined pollution of microplastics and other pollutants should be further accelerated.

Although this special issue reports some preliminary results on the interaction between microplastics and microorganisms, these findings are only a grain of corn in the wide sea. A number of new findings from other fields are not covered in this VSI, such as microplastics in indoor environments, urban pipe network, sewage treatment plants, and other artificial environments, as well as research in extreme environments such as the deep sea. In addition, the reported findings may be true only under some specific conditions, and some understandings are still matters of controversy. However, we are pleased to see that more and more of the public, and researchers, pay attention to the problem of microplastics, especially the in-depth crossover of multiple disciplines and the participation of more young researchers, which brings light to an early solution of this global environmental problem.

## Author Contributions

All authors listed have made a substantial, direct, and intellectual contribution to the work and approved it for publication.

## Conflict of Interest

The authors declare that the research was conducted in the absence of any commercial or financial relationships that could be construed as a potential conflict of interest.

## Publisher's Note

All claims expressed in this article are solely those of the authors and do not necessarily represent those of their affiliated organizations, or those of the publisher, the editors and the reviewers. Any product that may be evaluated in this article, or claim that may be made by its manufacturer, is not guaranteed or endorsed by the publisher.
